# Dynamic Alterations of the Intestinal Microbiota of Fifth-Instar Silkworms (*Bombyx mori*) Fed an Artificial Diet or Mulberry Leaves

**DOI:** 10.3390/insects15120970

**Published:** 2024-12-05

**Authors:** Chuanjie Chen, Meng Li, Feng Li, Xiaoyan Liang, Haiyang Zhang, Yinyu Gu, Guang Guo

**Affiliations:** 1 Shandong Institute of Sericulture, Shandong Academy of Agricultural Sciences, Yantai 265500, China; chuanjie79@163.com (C.C.); lim0320@126.com (M.L.); liangxiaoyan1001@163.com (X.L.); oceanz_zhy@163.com (H.Z.); 2 Shandong Engineering Research Center of Functional Crop Germplasm Innovation and Cultivation Utilization, Yantai 265500, China; 3 Shandong Academy of Agricultural Sciences, Jinan 250100, China; lfe1983@sina.com

**Keywords:** dynamic alterations, intestinal microbiota, silkworm, artificial diet, mulberry leaves

## Abstract

Intestinal microbes are known to impact the growth and development of insects. However, there are few reports on the intestinal microbiota of silkworms (*Bombyx mori*). The present study used Illumina 16S rRNA gene sequencing to investigate the changes over time in the intestinal bacteriome of fifth-instar silkworms fed mulberry leaf (MB) or artificial diet (AD). The results showed that the intestinal microbiota richness was significantly higher, before the 4th day of the fifth instar, in the silkworms fed AD rather than MB, while the richness was consistent between the AD and MB groups directly before cocooning. Proteobacteria was the most dominant phylum in MBs, AD, and the silkworm intestinal bacteriome, regardless of sex, feed type, or date, except that Firmicutes was the most dominant phylum for females on the 6th day of the fifth instar. *Acinetobacter* was the dominant genus in silkworms fed MB, while *Enterococcus* was the dominant genus in silkworms fed AD. Only 3.62% of the intestinal microbiota of silkworms fed MB was derived from MB, while 13.71% of the intestinal microbiota of silkworms fed AD was derived from AD. Thus, both bacterial communities were dominated by bacteria of unknown origin (non-feed sources). In the correlation network analysis, the silkworms fed AD appeared to have more complex interactions than the silkworms fed MB. Proteobacteria was the phylum most closely related to silkworm cocoon quality and feeding efficiency. *Pantoea* was the genera most closely related to cocoon quality and silkworm feeding efficiency in silkworms fed MB. AD had a significant impact on the predicted functions of the intestinal microbiota. There were significant differences in all six KEGG level 1 functions and all BugBase (except for Gram_Positive) phenotypes between silkworms fed AD or MB. The BugBase “Aerobic” phenotype was significantly higher in females compared to males, in both the AD and MB groups, while the “Oxidative_Stress_Tolerant” phenotype was the opposite. Overall, the findings suggest that the diversity, community structure, and predicted functions of intestinal bacteria in silkworms were significantly influenced by feed type. The study provides insights into the complex silkworm intestinal bacterial diversity and a foundation for probiotic screening.

## Simple Summary

Silkworms (*Bombyx mori*) have been bred by mulberry leaves for more than 5000 years. The use of an artificial diet frees producers from land- and season-related constraints, allowing year-round industrial cocoon production. However, the low cocoon yield prevents the application of an artificial diet. Intestinal microbes play an important role in host development and health, but little is known about gut bacteria associated with the silkworm. The present study focused on the dynamic alterations of the intestinal microbiota of fifth-instar silkworms (*Bombyx mori*) fed artificial diet and mulberry leaves, and aimed to explore the effect of the artificial diet on gut microbiota in silkworm and the dynamic interaction between silkworms and microorganisms, and screen potential probiotics. Overall, the findings suggest that the diversity, community structure, and predicted functions of intestinal bacteria in silkworms were significantly influenced by feed type.

## 1. Introduction

Silkworms (*Bombyx mori*), which are oligophagous Lepidoptera insects that can produce silk, have been bred by humans for more than 5000 years and have helped to shape world history through the famous Silk Road [1]. The mulberry leaf (MB) (*Morus* sp.) is the traditional food for silkworms. Due to the labor-intensive nature of the silk production industry, its competitiveness is gradually declining, but many attempts are being made to establish an artificial diet (AD) [2]. Since Fukuda et al. fed silkworms AD and succeeded in inducing silkworm larvae to produce 36 cocoons for the first time [3], AD has been used in silkworm research and silk production. Instead of relying entirely on cultivating mulberry trees to obtain feed, the use of AD frees producers from land- and season-related constraints, allowing year-round industrial cocoon production [4]. In addition, using AD reduces the labor intensity and so mitigates the difficulties facing this labor-intensive industry dealing with labor shortages [5]. Although the use of AD in the sericulture industry is very convenient, there are currently several problems associated with it, such as the low larval survival rate, the need to improve cocoon production, and multiple other issues affecting the various developmental stages [6].

An intestinal microecosystem is an important and complex biological system essential to the organisms’ health [7]. More and more research has confirmed that microbial symbionts, particularly those inhabiting the insect gut, are significant players in insect–plant interactions [8]. Intestinal microbes and insect hosts are interdependent, and the microbes play an important role in host development and health [9]. For instance, the intestinal microbes assist host insects to digest food [10,11], synthesize and absorb nutrients (such as amino acids, vitamins, and certain trace elements necessary for normal insect growth and development) [12,13], regulate the insect immune system to defend against pathogenic microbes, predators, and deleterious environmental factors [14,15], and improve insect host growth, development, and reproduction [16,17]. The ubiquitous nature of intestinal bacteria and increasing knowledge of their numerous advantages for insect hosts mean that they hold promise in multiple fields such as agriculture, ecology, medicine, and energy and environmental protection. For instance, *Lactobacillus* is the most commonly tested probiotic for use in insect feed [18]. *Bacillus* and *Pseudomonas* have also been developed as commercial biocontrol agents [19,20].

Like most insects, silkworm intestines are enriched with microbes that play crucial roles in their survival. Silkworms (*B. mori*) serve as model insects of the order Lepidoptera and are often employed for studying the relationships between microbes and hosts [21]. However, research on their intestinal microbiota remains limited [22]. Yeruva et al. identified potent probiotic bacteria from silkworm intestines through a metagenomic approach [23]. Liang et al. compared bacterial communities of silkworm larval intestines between traditionally reared silkworms and silkworms reared using a bioregenerative life support system [24]. Chen et al. reported that environmental factors, including diet and human manipulation during egg production practices, likely influence the silkworm intestinal microbiota composition [25]. Dong et al. analyzed the differences between the intestinal microbiota of fifth-instar larvae fed MB or AD, and they found that the microbial diversity was lower in the latter [26]. Liu et al. demonstrated the importance of intestinal microbes in silkworm defense against viral pathogens [27]. Li et al. demonstrated the relationship between the intestinal microbiota and fluoride resistance of silkworms [28]. Gunasekhar and Somayaji demonstrated the positive effect of *Burkholderia cepacia* on silkworm growth and enzymatic activity [29].

Nutrient absorption and disease in silkworms are both closely related to the larval midgut microbiota [30]. The fifth instar is a key stage of silkworm development, as the amount of MB ingested at this stage is about 85% of the total amount across all larval stages [31]. Furthermore, the microbiota composition and structure are dynamic, varying with nutrient availability, physiological environment, and proximity to other organisms [32]. Berg et al. also reported that temporal and spatial microbiota changes are important for understanding microbiota function [33]. Diet has been shown to be one of the most important factors that alter insect physiological activity and the intestinal microbiota [24]. Understanding the changes in intestinal bacteria over time should help to improve silkworm health and nutrient absorption. To our knowledge, there are no reports on the changes over time in the intestinal microbiota of fifth-instar silkworms fed MB or AD. Thus, this study aimed to explore these changes, and also investigate the intestinal microbiota diversity and functions, the associations of the intestinal microbiota with silkworm feeding efficiency and cocoon quality, and the complex interactions of the intestinal microbes with their silkworm hosts.

## 2. Materials and Methods

### 2.1. AD and MB Preparation

The AD was prepared by our team (Shandong Institute of Sericulture, Shandong Academy of Agricultural Sciences). It comprised 38% MB powder, 38% soybean powder, 6% MB green twig powder, 12.4% starch, 1.5% vitamin C, 1.5% vitamin B complex, 2.4% citric acid, and 0.2% choline chloride. This powder was mixed thoroughly with 1.9 times (*w*/*w*) water, boiled at 100 °C for 40 min, cooled naturally, and stored at 4 °C.

Fresh MB was picked from mulberry trees (Xuan 792 strain) at the experimental farm of Shandong Sericulture Research Institute (37°08′25.44″ N, 121°08′33.98″ E) in Yantai, Shandong Province, China.

### 2.2. Silkworm Rearing

Silkworms (*B. mori* strain Jingsong × Haoyue) were fed fresh MB (*Morus* L.) until the 4th instar. The conditions were 26 ± 1 °C, 70 ± 15% humidity, and a 12/12 h light/dark photoperiod with a fresh air system.

The 5th-instar silkworms, fed MB (MBs) and fed AD (ADs), were grouped into the following groups (three replicates in each group): female silkworms fed MB (MB_S_f_), male silkworms fed MB (MB_S_m_), female silkworms fed AD (AD_S_f_), and male silkworms fed AD (AD_S_m_).

The intestinal microbiota of the 5th-instar silkworms were also analyzed (and compared to the microbiota in the MB feed and AD feed) based on the following groups: intestinal microbiota of silkworms fed MB (MB_I_), intestinal microbiota of silkworms fed AD (AD_I_), intestinal microbiota of male silkworms fed MB (MB_I_m_), intestinal microbiota of female silkworms fed MB (MB_I_f_), intestinal microbiota of male silkworms fed AD (AD_I_m_), intestinal microbiota of female silkworms fed AD (AD_I_f_), intestinal microbiota of 1st-day silkworms of 5th instar fed MB (MB_I_1_), intestinal microbiota of 4th-day silkworms of 5th instar fed MB (MB_I_4_), intestinal microbiota of 6th-day silkworms of 5th instar fed MB (MB_I_6_), intestinal microbiota of 1st-day silkworms of 5th instar fed AD (AD_I_1_), intestinal microbiota of 4th-day silkworms of 5th instar fed AD (AD_I_4_), and intestinal microbiota of 6th-day silkworms of 5th instar fed AD (AD_I_6_).

### 2.3. Assessment of Silkworm Feeding Efficiency and Silkworm Cocoon Quality

To assess silkworm feeding efficiency, the amount of ingested food, amount of digested food, and digestion rate were calculated. The MB and AD before and after feeding the silkworms were weighed regularly every day, as was the excrement. Leftover leaves and excrement were dried in a hot air oven daily at about 100 °C until they reached constant weight to assess the moisture content. Ingesta (g) = Dry weight of food fed − Dry weight of leftover food. Digesta (g) = Dry weight of food ingested − dry weight of litter. Digestibility (%) = (Dry weight of Digesta/Dry weight of Ingesta) × 100.

To assess silkworm cocoon quality, the whole cocoon weight, cocoon shell weight, and cocoon shell rate were assessed on the 7th day after silkworm spinning.

### 2.4. Intestinal Bacterial Community Analysis

For the intestinal bacterial community analyses, the excrement was taken on the 2nd, 5th, and 7th days of the 5th instars, and frozen at −80 °C immediately. Genomic DNA was extracted from the silkworm excrement using an E.Z.N.A.^®^ Soil DNA Kit (Omega Bio-Tek, Norcross, GA, USA). The bacterial universal V3-V4 region of the 16S rRNA gene was amplified by polymerase chain reaction (PCR) using primers 338F (5′-ACTCCTACGGGAGGCAGCAG-3′) and 806R (5′-GGACTACHVGGGTATCTAAT-3′) (see Table S1 for DNA metabarcoding details). The PCR products were quantified using a Quantus™ Fluorometer (Promega Corporation, Madison, WI, USA) after purification. The purified amplicons were mixed in equimolar amounts and sequenced by Majorbio Bio-Pharm Technology Co., Ltd. (Shanghai, China) on an Illumina MiSeq PE300 platform (Illumina Inc., San Diego, CA, USA). More details on the DNA metabarcoding analyses can be found in Table S1. All sequences have been deposited in the National Center for Biotechnology Information (NCBI) Sequence Read Archive database (accession no. PRJNA1128353).

### 2.5. Data Analysis

Calculations were performed using Microsoft Excel, and statistical analyses were performed using DPS Statistics v18.10 (http://www.dpsw.cn, accessed on 3 December 2024). Some analyses were also conducted on the Majorbio Cloud Platform (www.majorbio.com, accessed on 3 December 2024); α-diversity was calculated and rarefaction curves (at a 97% identity level) were generated using Mothur v1.30.2; Venn diagrams and bar charts were generated using R script v3.3.1. β-diversity was assessed using principal coordinate analysis (PCoA) based on the Bray–Curtis distance matrix.In addition, redundancy analysis was conducted and visualized using the rda and vegan packages in R v3.3.1, respectively. SourceTracker v1.0.1 was used to explore the source ratio of each sink sample [34]. The RandomForest and plotROC packages in R v3.3.1 were used to conduct random forest analysis and receiver operating characteristic (ROC) curve analysis to calculate the area under the curve (AUC), respectively. Finally, network analysis was performed using NetworkX (version1.11) software. Bacterial phenotypes were predicted using BugBase (https://bugbase.cs.umn.edu/index.html, accessed on 3 December 2024) [35], which relies on PICRUSt (http://huttenhower.sph.harvard.edu/galaxy, accessed on 3 December 2024), IMG (http://img.jgi.doe.gov, accessed on 3 December 2024), KEGG (http://www.genome.jp/kegg/, accessed on 3 December 2024), and PATRIC https://patric.vbi.vt.edu, accessed on 3 December 2024) tools. Data are presented as mean ± standard error. Differences among the means of different groups were considered significant at *p* < 0.05 using the Duncan test.

## 3. Results

### 3.1. Effects of MB and AD on Silkworm Feeding Efficiency and Silkworm Cocoon Quality

**Figure 1:**
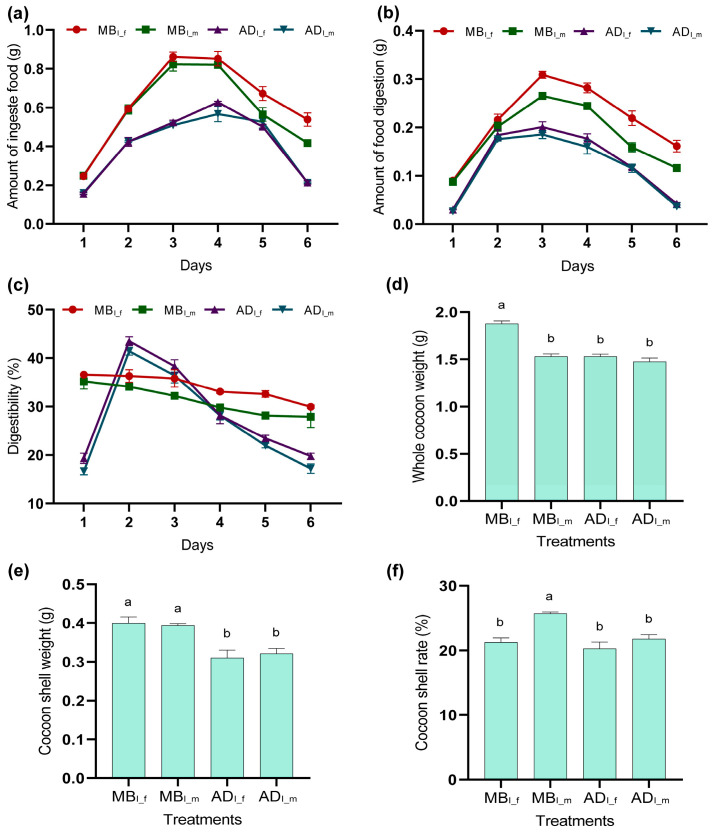
Feeding efficiency and cocoon quality of fifth-instar silkworms. MB_I_f_: female silkworms fed mulberry leaves. MB_I_m_: male silkworms fed mulberry leaves. AD_I_f_: female silkworms fed artificial diet. AD_I_m_: male silkworms fed artificial diet. (**a**) Amount of ingested food. (**b**) Amount of digested food. (**c**) Digestion rate. (**d**) Whole cocoon weight. (**e**) Cocoon shell weight. (**f**) Cocoon shell rate. Different lowercase letters indicate significant differences between groups (*p* < 0.05).

The differences in feeding efficiency and cocoon quality of MB_S_ vs. AD_S_ are summarized in Figure 1. The amounts of ingested and digested food were higher for MB_S_ vs. AD_S_ (Figure 1a,b). The digestion rate was highest on the 1st day (of the fifth instar) for MB_S_, and the 2nd day for AD_S_.

The whole cocoon weight was significantly higher for MB_S_f_ vs. MB_S_m_ (Figure 1d). The cocoon shell weight was significantly higher for MB_S_ vs. AD_S_ (Figure 1e). Lastly, the cocoon shell rate was highest for MB_S_m_ (Figure 1f). The photos of silkworms and cocoons are shown in Figure S1.

### 3.2. Bacterial Compositionand α-Diversity

The rarefaction curve of the 42 samples approached a saturation plateau (Figure S2), suggesting that the data set was large enough to fully reflect the bacterial diversity. The number of OTUs was highest in AD and lowest in MB_I_m6_. Richness was higher in AD vs. MB. Richness were significantly higher in AD_I_ vs. MB_I_ before the 4th day of the fifth instar. Richness was non-significantly higher in females vs. males, for both AD_I_ and MB_I_. Richness was significantly higher in AD_I_1_ and AD_I_4_ vs. AD_I_6_, but only non-significantly higher in MB_I_1_ vs. MB_I_6_. Richness in males, for both MB_I_ and AD_I_, non-significantly decreased with day age (of the fifth instar). α-diversity exhibited similar patterns to richness (Table S2).

The numbers of shared and unique bacterial OTUs in the different groups are shown in Figure S3.

Regarding MB and AD, of the 632 total OTUs, only 59 were shared by MB and AD (Figure S3a). Regarding MB_I_ and AD_I_, of the 1062 total OTUs, 275 were shared by MB_I_ and AD_I_ (Figure S3b). The numbers of unique OTUs were much higher in AD vs. MB, and AD_I_ vs. MB_I_.

Regarding MB and all MB_I_ groups, of the 424 total OTUs, 40 were shared by MB, MB_I_f_, and MB_I_m_ (Figure S3c), 30 by MBs, MB_I_1_, MB_I_4_, and MB_I_6_ (Figure S3e), and 16 by all groups (Figure S3g); the number of unique OTUs was highest for MB_I_f_6_.

Regarding AD and all AD_I_ groups, of the 1312 total OTUs, 160 were shared by AD, AD_I_f_, and AD_I_m_ (Figure S3d), 122 by AD, AD_I_1_, AD_I_4_, and AD_I_6_ (Figure S3f), and 56 by all groups (Figure S3h); the number of unique OTUs was highest for AD.

### 3.3. Taxonomic Analysis of Intestinal Microbiota

**Figure 2:**
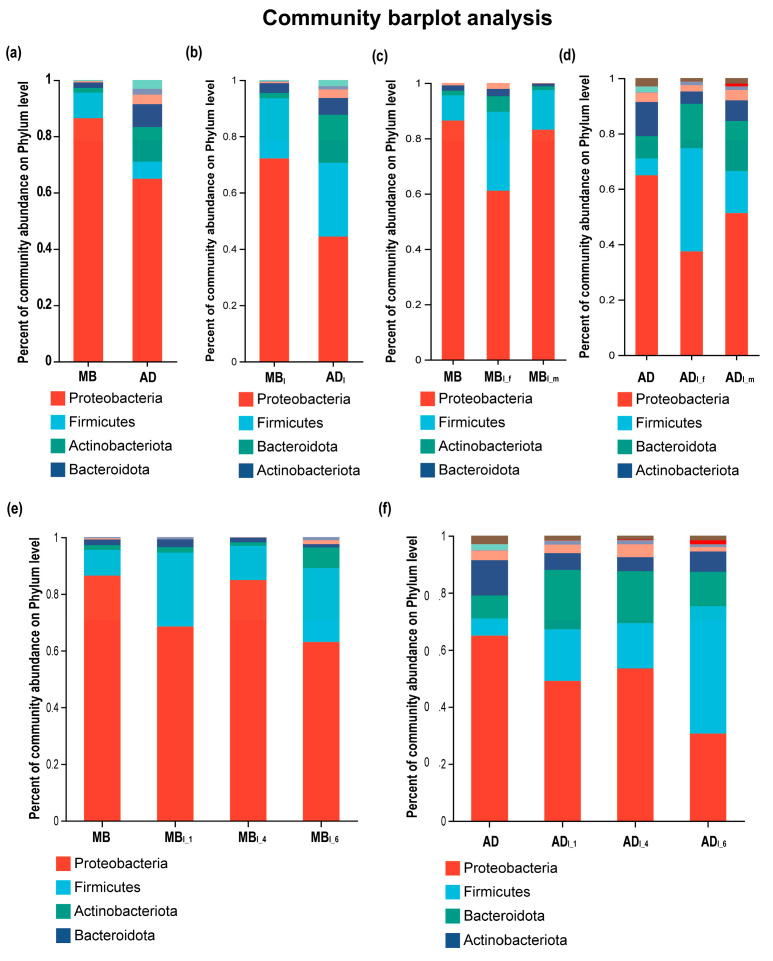
Relative abundances of bacteria at the phylum level in each group. MB: mulberry leaf. AD: artificial diet. MB_I_: intestinal microbiota of silkworms fed mulberry leaves. AD_I_: intestinal microbiota of silkworms fed artificial diet. MB_I_f_: intestinal microbiota of female silkworms fed mulberry leaves. MB_I_m_: intestinal microbiota of male silkworms fed mulberry leaves. AD_I_f_: intestinal microbiota of female silkworms fed artificial diet. AD_I_m_: intestinal microbiota of male silkworms fed artificial diet. MB_I_1_: intestinal microbiota of 1st-day silkworms of fifth instar fed mulberry leaves. MB_I_4_: intestinal microbiota of 4th-day silkworms of fifth instar fed mulberry leaves. MB_I_6_: intestinal microbiota of 6th-day silkworms of fifth instar fed mulberry leaves. AD_I_1_: intestinal microbiota of 1st-day silkworms of fifth instar fed artificial diet. AD_I_4_: intestinal microbiota of 4th-day silkworms of fifth instar fed artificial diet. AD_I_6_: intestinal microbiota of 6th-day silkworms of fifth instar fed artificial diet. The following figures use the same abbreviations. (**a**) MB and AD. (**b**) MB_I_ and AD_I_. (**c**) MB, MB_I_f_, and MB_I_m_. (**d**) AD, AD_I_f_, and AD_I_m_. (**e**) MB, MB_I_1_, MB_I_4_, and MB_I_6_. (**f**) AD, AD_I_1_, AD_I_4_, and AD_I_6_.

The 1432 OTUs were classified into 39 phyla, 102 classes, 237 orders, 387 families, 746 genera, and 1117 species. At the bacterial phylum level (Figure 2), Proteobacteria (86.47%) and Firmicutes (9.06%) were the most dominant in MB, while Proteobacteria (64.93%) and Actinobacteriota (12.33%) were the most dominant in AD (Figure 2a). Proteobacteria decreased and Firmicutes increased in MB_I_ vs. MB, and AD_I_ vs. AD. Proteobacteria followed by Firmicutes were the most dominant in both MB_I_ and AD_I_ (Figure 2b). Proteobacteria was higher and Firmicutes was lower in males vs. females, for both MB_I_ and AD_I_ (Figure 2c,d). Additionally, Proteobacteria was higher in MB_I_4_ vs. MB_I_1_ or MB_I_6_, and AD_I_4_ vs. AD_I_1_ or AD_I_6_.

There were significant differences in the dominant bacterial genera in MB vs. AD. *Pantoea* (accounting for 48.66% of bacteria) followed by *Pseudomonas* were the dominant genera in MB, compared to *Acinetobacter*, followed by *Aeromonas* in AD (Figure S4a). *Enterobacter* and *Staphylococcus* were the dominant genera in MB_I_, compared to *Acinetobacter* and *Enterococcus* in AD_I_ (Figure S4b). *Enterobacter* and *Staphylococcus* were the dominant genera in MB_I_f_, compared to *Enterobacter* and *Pantoea* in MB_I_m_ (Figure S4c). *Enterococcus* and *Acinetobacter* were the dominant genera in AD_I_f_, compared to *Acinetobacter* and *Chryseobacterium* in AD_I_m_ (Figure S4d). *Enterobacter* and *Pantoea* were the dominant genera in MB_I_1_, compared to *Enterobacter* and *Acinetobacter* in MB_I_4_, and compared to *Enterobacter* and *Staphylococcus* in MB_I_6_ (Figure S4e). *Acinetobacter* and *Chryseobacterium* were the dominant genera in both AD_I_1_ and AD_I_4_, compared to *Enterococcus* and *Weissella* in AD_I_6_ (Figure S4f).

**Figure 3:**
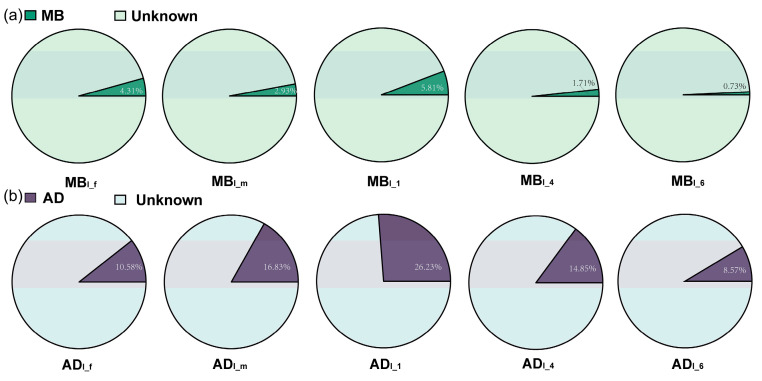
SourceTracker analysis of silkworm intestinal microbiota. (**a**) MB_I_. (**b**) AD_I_.

The SourceTracker analysis is shown in Figure 3. MB_I_ and AD_I_ were identified as the sink samples, and MB and AD were identified as the sources. MB and AD represented the origins of 3.62% and 13.71% of bacteria in MB_I_ and AD_I_, respectively. Thus, the bacterial communities of MB_I_ and AD_I_ were dominated by bacteria of unknown origin, indicating that the silkworm intestinal microbiota mainly came from the environment rather than diet. The bacterial community of AD_I_ was more derived from diet than that of MB_I_. The proportion of bacteria derived from diet decreased with day age (of the fifth instar).

### 3.4. β-Diversity Analysis

PCoA was conducted to further compare the bacterial communities of MB_I_ vs. AD_I_ (Figure S5). This analysis revealed the main variations in bacterial community composition and abundance among the groups. The PC2 value was higher for MB vs. AD (Figure S5a), while the PC1 value was lower for MB_I_ vs. AD_I_ (Figure S5b). In addition, both sex and day age (of the fifth instar) significantly affected the bacterial community composition of MB_I_ (Figure S5c,e), but only day age (of the fifth instar) significantly affected that of ADI (Figure S5d,f).

### 3.5. Differential Bacterial Taxa

**Figure 4:**
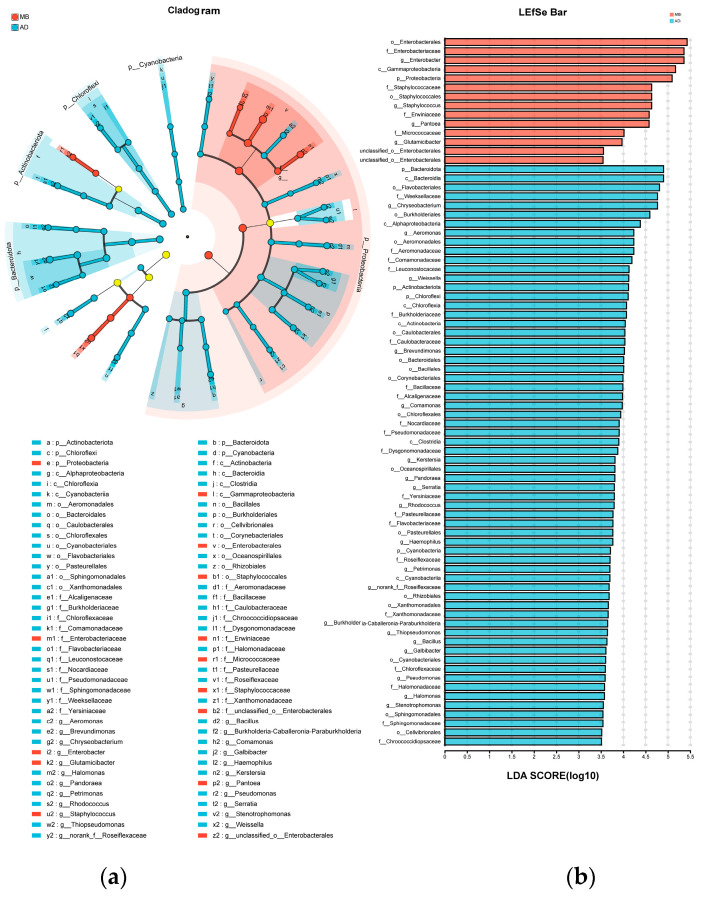
Linear discriminant analysis effect size (LEfSe) and linear discriminant analysis (LDA) of silkworm intestinal microbiota. (**a**) LEfSe. (**b**) LDA.

Figure 4 shows the taxa that significantly differentiated MB_I_ vs. AD_I_, based on linear discriminant analysis effect size (LEfSe). For MB_I_, one phylum, one class, four orders, four families, and four genera were enriched. For AD_I_, 4 phyla, 6 classes, 15 orders, 20 families, and 19 genera were enriched. The genera that significantly differentiated MB_I_ vs. AD_I_ mostly (more than half) belonged to Proteobacteria, followed by Bacteroidota.

**Figure 5:**
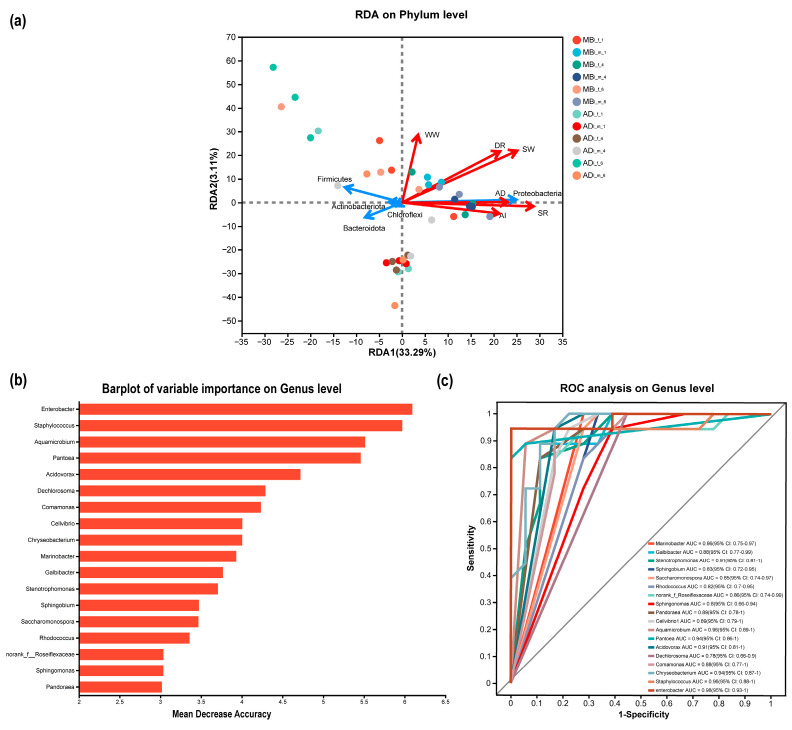
Redundancy and random forest analyses. (**a**) Redundancy analysis. (**b**) Random forest analysis. (**c**) ROC curve.

Figure 5b shows the key characteristic genera (biomarkers) involved in the differential formation of MB_I_ vs. AD_I_, based on a random forest analysis. The 18 most important and reliable genera (including *Enterobacter*, *Staphylococcus*, and *Pantoea*) were selected based on ten-fold cross validation. A ROC curve analysis was used to test the accuracy of the random forest prediction (Figure 5c). The AUC value of the 18 genera was 0.97, indicating a good classification performance. These 18 genera, as potential bacterial biomarkers, might significantly differentiate MB_I_ vs. AD_I_.

### 3.6. Correlation Network Analyses

**Figure 6:**
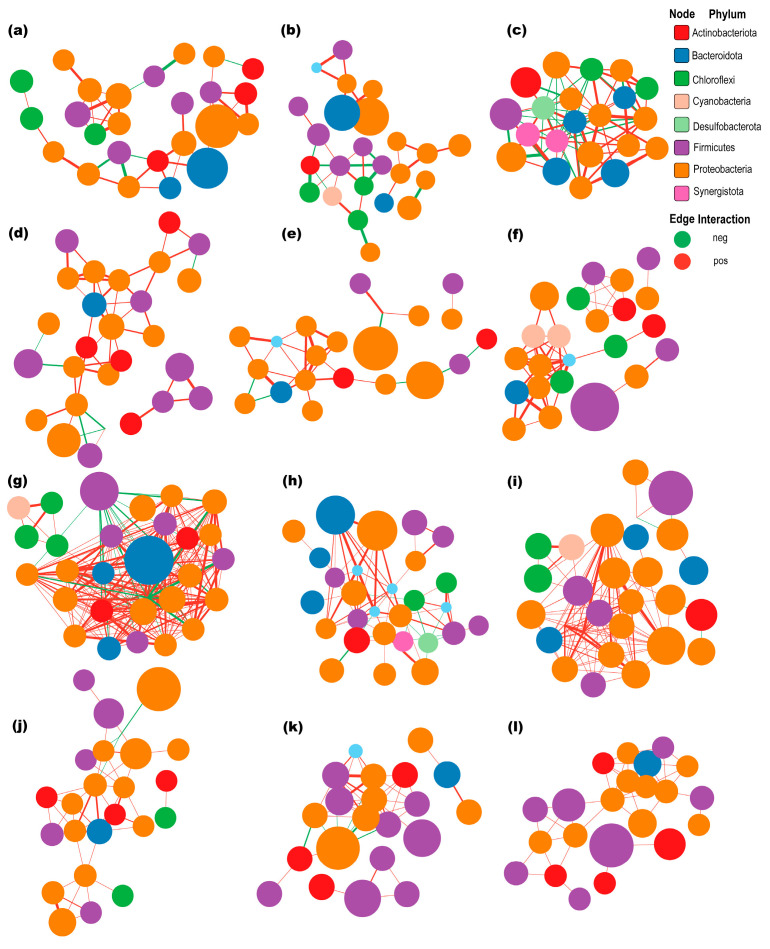
Correlation network analyses of bacterial communities. (**a**) AD_I_1_. (**b**) AD_I_4_. (**c**) AD_I_6_. (**d**) MB_I_1_. (**e**) MB_I_4_. (**f**) MB_I_6_. (**g**) AD_I_f_. (**h**) AD_I_m_. (**i**) AD_I_. (**j**) MB_I_f_. (**k**) MB_I_m_. (**l**) MB_I_.

To explore the complexity of the interactions in the bacterial communities in the different groups, correlation network analyses were conducted. This revealed that the correlations were more complex for AD_I_ vs. MB_I_. Specifically, the average degree and numbers of positive and negative edges were all higher for AD_I_ (average degree = 8.36, positive edges = 113, negative edges = 4) (Figure 6i) vs. MB_I_ (average degree = 4.45, positive edges = 49, negative edges = 0) (Figure 6l).

Additionally, for both AD_I_ and MB_I_, the complexity and modular structure were higher on the 6th day (Figure 6c,f) vs. 1st day (Figure 6a,d) and 4th day (Figure 6b,e). Specifically, the average degree was higher on the 6th day (average degree = 7.36 for AD_I_, and 4.17 for MB_I_) vs. 1st day (average degree = 2.96 for AD_I_, and 3.33 for MB_I_) and 4th day (average degree = 2.58 for AD_I_, and 2.76 for MB_I_) (Table S3). Furthermore, the numbers of positive and negative edges were higher for AD_I_6_ (positive edges = 46, negative edges = 35) vs. AD_I_1_ and AD_I_4_.

Moreover, the average degree and the numbers of positive and negative edges were higher for AD_I_f_ (average degree = 16.13, positive edges = 209, negative edges = 33) (Figure 6g) vs. AD_I_m_ (average degree = 4.86, positive edges = 57, negative edges = 11) (Figure 6h), but there were no differences for MB_I_m_ vs. MB_I_f_ (Figure 6j,k). Additionally, there were more positive than negative edges in all groups (Table S3).

### 3.7. Associations of Bacteria Community Composition with Silkworm Cocoon Quality and Silkworm Feeding Efficiency

The redundancy analysis revealed the associations of bacterial community composition with amount of ingested food, amount of digested food, digestion rate, whole cocoon weight, cocoon shell weight, and cocoon shell rate (Figure 5a). The first axis accounted for 33.29% of the overall variation in bacterial community composition, while the second axis accounted for only 3.11%. The redundancy analysis showed that the MB_I_ bacterial community structure was positively correlated with cocoon shell rate and cocoon shell weight, and Proteobacteria was positively correlated with cocoon shell rate, amount of ingested food, and amount of digested food.

**Figure 7:**
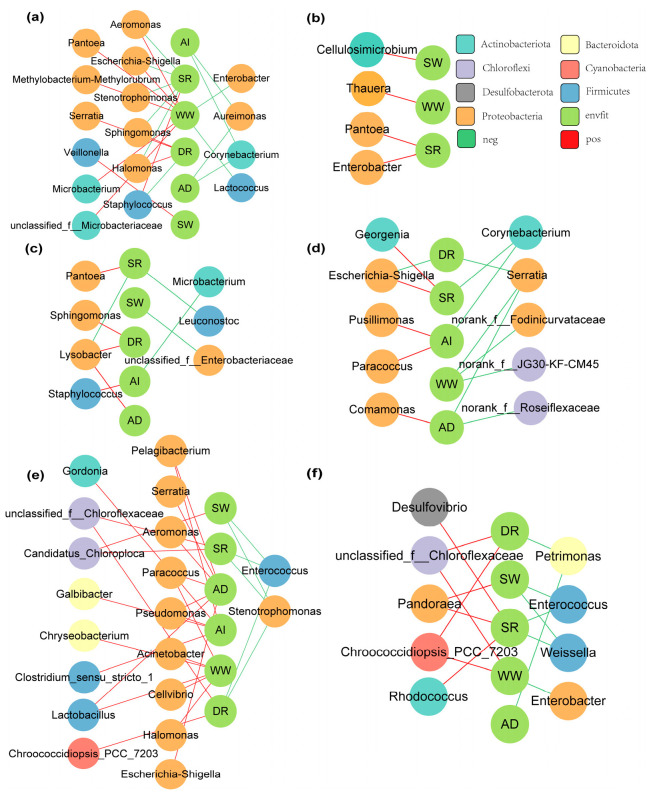
Correlation network analyses of bacterial communities and both silkworm feeding efficiency and cocoon quality (**a**) MB_I_1_. (**b**) MB_I_6_. (**c**) MB_I_4_. (**d**) AD_I_1_. (**e**) AD_I_4_. (**f**) AD_I_6_.

Correlation network analyses of the bacterial communities and both silkworm feeding efficiency and silkworm cocoon quality were conducted to study the interactions (Figure 7). The bacterial community complexity was greater for AD_I_ vs. MB_I_, for MB_I_1_ vs. MB_I_4_ or MB_I_6_, and for AD_I_4_ vs. AD_I_1_ or AD_I_6_. Proteobacteria was the phylum most closely related to silkworm cocoon quality and feeding efficiency for both MB_I_ and AD_I_. *Pantoea* was the genus most closely related to silkworm cocoon quality and silkworm feeding efficiency for MB_I,_ while *Rhodococcus, Pandoraea, g__unclassified_f__Chloroflexaceae*, and *Chroococcidiopsis_PCC_7203* were the genera most closely related to silkworm cocoon quality and silkworm feeding efficiency for AD_I_ (Figure 7). The complexity of MB_I_, but not AD_I_, decreased with increasing day age (of the fifth instar) (Table S4).

### 3.8. Predicted Functional Consequences

The functions of the bacterial communities in all groups were predicted using six KEGG level 1 functions (Metabolism, Genetic Information Processing, Environmental Information Processing, Cellular Processes, Organismal Systems, and Human Diseases) (Figure S6). The relative abundances of all six functions were significantly higher in AD_I_ vs. MB_I_, which indicated that AD had a large impact on the intestinal microbiota functions. Regarding AD_I_m_ and AD_I_f_, Cellular Processes on the 4th and 6th days and Organismal Systems on the 6th day were significantly higher in males. Regarding MB_I_m_ and MB_I_f_, there were significant differences in Environmental Information Processing and Cellular Processes on the 4th day and Genetic Information Processing on the 6th day. In addition, Metabolism was far more abundant than the other functions (Figure S6 and Table S5). Significant differences between AD_I_ and MB_I_ with respect to hormone-related functions are shown in Figure S7, with AD_I_ significantly higher than MB_I_.

**Figure 8:**
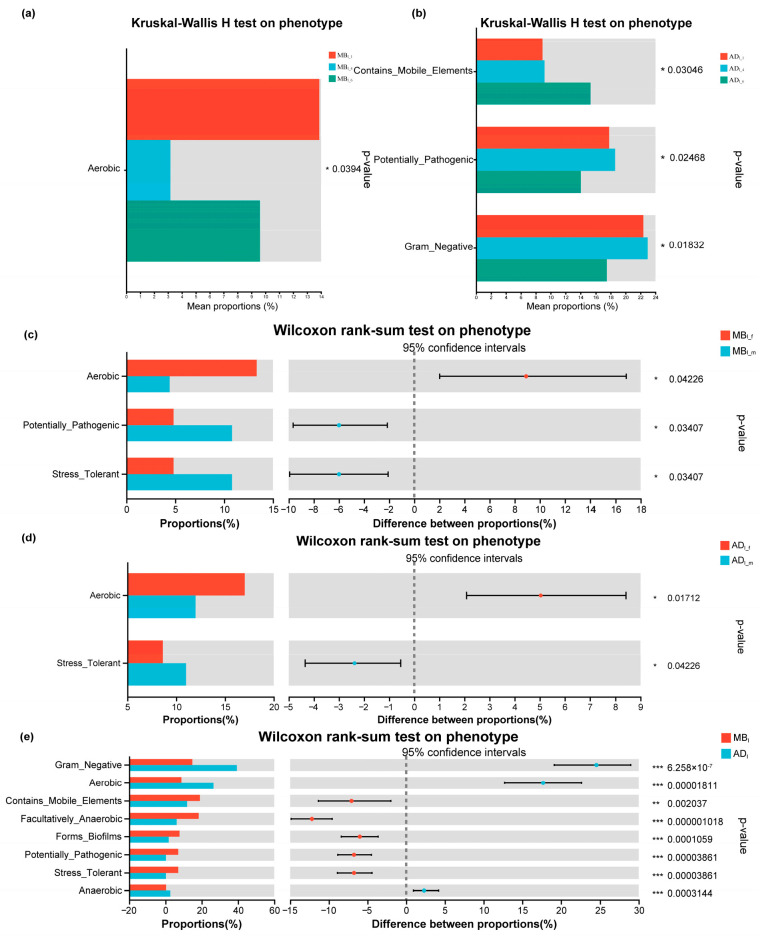
BugBase functional prediction of silkworm intestinal microbiota. (**a**) MB_I_1_, MB_I_4_, and MB_I_6_. (**b**) AD_I_1_, AD_I_4_, and AD_I_6_. (**c**) MB_I_f_ and MB_I_m_. (**d**) AD_I_f_ and AD_I_m_. (**e**) MB_I_ and AD_I_. *** indicates a signiffcant difference at *p* < 0.001,  ** *p* < 0.01,  * *p* <0.05.

The BugBase algorithm predicts organism-level functional pathways and biologically interpretable phenotypes (Gram Positive, Gram Negative, Biofilm Forming, Pathogenic, Mobile Element-Containing, Aerobic, Anaerobic, Facultatively Anaerobic, and Oxidative-Stress Tolerant) using whole-genome shotgun or marker gene sequencing data [35]. There were significant differences between AD_I_ and MB_I_, except for Gram_Positive. Specifically, Gram_Negative, Anaerobic, and Aerobic were significantly higher in AD_I_ vs. MB_I_; however, the other five BugBase phenotypes were significantly higher in MB_I_ vs. AD_I_ (Figure 8e). The phenotypes Contains_Mobile_Elements, Potentially_Pathogenic, and Gram_Negative significantly differed by day age for AD_I_, while only Aerobic significantly differed by day age for MB_I_ (Figure 8a,b). Aerobic was significantly higher in females vs. males for both AD_I_ and MB_I_ while Oxidative_Stress_Tolerant was the opposite. Potentially_Pathogenic was significantly higher in males vs. females for MB_I_, but there was no significant difference for AD_I_ (Figure 8c,d).

## 4. Discussion

The amount of food consumed by larvae affects their growth and development [36]. Additionally, the cocoon yield traits (whole cocoon weight, cocoon shell weight, and cocoon shell rate) have drawn much attention [37], as they are closely related to sericulture profits. The cocoon quality was superior in MB_S_ vs. AD_S_, and males vs. females, which was well known [38,39,40,41]. Feeding efficiency was also superior in MB_S_ vs. AD_S_, which was consistent with research by Yin [41]. The highest digestion rate occurred on the 1st day (of the fifth instar) for MB_S_, which was consistent with Anantha Raman et al. [42], while it occurred on the 2nd day for AD_S,_ which was inconsistent with the 1st day considered by Horie et al. [43]. Many factors affect feeding efficiency (such as feed formula, feeding environment, and silkworm variety), the silkworm in this study were fed mulberry leaf before the fourth instar; the adaptation process might occurred when transferred to artificial diet at fifth instar, as for specific reasons for the difference in digestion rate in MB_S_ vs. AD_S_ deserves further investigation.

The richness was significantly higher in AD_I_ vs. MB_I_ before the 4th day of the fifth instar, which differed from previous research [26,44]. The different results may be due to differences in feed, silkworm varieties, or environment. In particular, the results of the present study may be attributable to the significantly higher richness of AD vs. MB, potentially because AD contained soybean powder, starch, etc. in addition to MB. In addition, AD was sterilized and then stored in a refrigerator for 6 days throughout the entire fifth-instar stage without further sterilization, which may have led to an increase in environmental bacteria.

The richness was significantly lower over time in AD_I_ (in AD_I_6_ vs. AD_I_1_ and AD_I_4_). This suggests that when silkworms (especially males) are fed a large number of bacteria, they may begin to negatively regulate the bacteria in their bodies, thus reducing the bacteria in their excrement. Shu et al. also reported that the microbial populations tended to be simpler over time (in the 3rd vs. 1st and 2nd instars) in the AD group, though they did not report on the fifth instars [21]. Richness was also lower in MB_I_6_ vs. MB_I_1_, but the difference was nonsignificant, which may be due to the low richness of MB. The decreased richness on the 6th day in both AD_I_ and MB_I_ may also be due to the silkworms being about to cocoon (and thus reducing their intake and emptying their digestive tracts). Furthermore, the SourceTracker analysis showed that MB and AD represented the origins of 3.62% and 13.71% of bacteria in MB_I_ and AD_I_, respectively, which indicated that non-feed sources were the main sources of the silkworm intestinal microbiota.

Previous research showed that the silkworm intestinal microbiota composition varied with diet and other factors [31]. The top three dominant phyla were reported to be Cyanobacteria, Firmicutes, and Proteobacteria [26], or Proteobacteria, Actinobacteria, and Firmicutes [44], in both MB_I_ and AD_I_, though the proportions differed slightly between the groups. In the present study, Proteobacteria, Actinobacteria, and Firmicutes were the dominant bacterial phyla in MB_I_, while Proteobacteria, Firmicutes, and Bacteroidota were the dominant bacterial phyla in AD_I_. The differences between studies may have been caused by regional differences. Cyanobacteria, which had the highest abundance in the study by Dong et al., was almost absent in the present study [26]. Additionally, both the redundancy analysis and correlation network analysis showed that Proteobacteria was positively correlated with cocoon quality.

Furthermore, the abundances of dominant genera significantly differed in AD_I_ vs. MB_I_. In the study by Dong et al., *Streptophyta(o)*, *Enterococcus*, and *Pseudomonas* (MB_I_), and *Enterococcus*, *Streptophyta(o)*, and *Pseudomonas* (AD_I_) were the most abundant [26]. In the study by Qin et al., *Ralstonia*, *Rhodococcus*, and *Burkholderia–Caballeronia–Paraburkholderia* (AD_I_), and *Burkholderia–Caballeronia–Paraburkholderia*, *Ralstonia*, and *Rhodococcus* (MB_I_) were the most abundant [44]. In the present study, *Enterobacter*, *Staphylococcus*, and *Acinetobacter* (MB_I_), and *Enterococcus*, *Acinetobacter*, and *Chryseobacterium* (AD_I_) were the most abundant. The differences among these studies may be caused by factors such as silkworm variety, feed type, or environment, and especially environment, as the silkworm intestinal microbiota mainly comes from the environment, based on the SourceTracker analysis. Based on the above, the dominant bacterial genera in MB varied greatly by region (dominant bacteria in MB from one region were almost undetectable in MB from another region; e.g., *Streptophyta* was not found in our study). In addition, the high similarity between MB_I_ and AD_I_ in same region may be due to the fact that the mulberry leaf powder used in the artificial diet generally came from the local MB. Nevertheless, none of the dominant bacterial genera in MB or AD had serious pathogenicity regarding silkworms.

Key differential bacteria, including: *Enterococcus*, *Enterobacter*, *Staphylococcus*, *Pantoea*, *Acinetobacter* and *Chryseobacterium*, between AD_I_ and MB_I_ were identified in a random forest analysis. All these genera, except for *Enterococcus*, were positively correlated with cocoon quality. However, further research is needed to determine which species are probiotics.

*Enterococcus* was much lower in AD vs. MB, but much higher in AD_I_ vs. MB_I_, which may contribute to the lower intestinal pH of AD_S_ vs. MB_S_. The abundance of *Enterococcus* is related to the immune system response [26]. *Enterococcus* is abundant in the silkworm larvae digestive tract [26,45], which may decrease the intestinal pH via acetate production, protect the host against certain toxins via physical and chemical mechanisms, and contribute to disease resistance by inhibiting the germination of the fungus *Nosema bombycis* [24,46,47]. Kumar et al. speculated that the substantially higher abundance of *Enterococcus* in wild silkworm (*B. mandarina*) vs. domesticated silkworm (*B. mori*) may be caused by the direct exposure of the former to the natural environment [45]. Although *Enterococcus* has efficient L-tryptophan production and probiotic potential for silkworms [48], there is also a report of *Enterococcus* spp. being opportunistic silkworm pathogens [45]. In the present study, *Enterococcus* was negatively related with both cocoon quality and feed efficiency when fed AD, and the relative abundance was significantly higher in AD_I_ vs. MB_I_, which means that the *Enterococcus* in this study still had negative effects, despite not being pathogenic, as did *Weissella*. Whether *Enterococcus* is a probiotic or pathogen may depend on the different functions of different species of *Enterococcus*.

*Enterobacter* was much higher in MB_I_ vs. AD_I_, which may be related to the digestion of the higher crude-fiber content in MB (Figure S8). *Enterobacter* (isolated from *B. mori*) was shown to utilize various polysaccharides, including cellulose [49], and *Enterobacter* also generates hydrogen via waste fermentation [50,51], produces exopolysaccharide [52], and fixes nitrogen [53]. *Enterobacter* has been reported to be a probiotic for mass Mediterranean fruit fly rearing for sterile insect technique applications [54].

*Staphylococcus* was lower in MB_I_m_ vs. MB_I_f_, as also reported by [30], and this deserves further exploration. *Staphylococcus* can be a pathogen [55] and produces lipase [56]. However, *Staphylococcus* was the most dominant genus (29%) in honey bees, and it was considered to be a beneficial intestinal inhabitant and to be involved in the maintenance of a healthy microbiota, as it is tolerant to acidic environments and helps to ferment sugars [57].

*Pantoea* was much higher in MB vs. AD, and MB_I_ vs. AD_I_, which may be related to the higher crude-fiber content in MB. Additionally, *Pantoea* was positively correlated with cocoon shell rate, indicating that it may be beneficial for silkworm cocoon production. The intestinal bacterial genus *Pantoea* aids nitrogen-fixing leaf-cutter ants [58]. Another study revealed that *Pantoea* and *Klebsiella* contained several sequences predicted to code for enzymes (including cellulases, β-galactosidases, chitinases, α-rhaxylosidases, α-mannosidases, α-rhamnosidases, and pectinesterases) that supported the digestion, decomposition, and absorption of food plants, and these bacteria, as persistent cellulose-degrading bacterial symbionts, may work with fungi to deconstruct plant polymers [59]. Ling et al. also reported that *Pantoea* was significantly positively correlated with cellulose digestibility [60]. *Pantoea agglomerans* was also found to release large amounts of guaiacol and small amounts of phenol, both of which are components of the locust cohesion pheromone [61].

*Acinetobacter* was the genus with the highest probability of ranking in the top three among all groups (Table S3). It also decreased sharply on the 6th day of the fifth instar, which may be related to the sharp decrease in silkworm feed consumption on this day. *Acinetobacter* is a dominant genus typically found in insect intestines [62], and it plays important roles, contributing to nutrient utilization, life cycle completion, and protection against host-plant secondary metabolites and unstable environmental conditions [63]. Increased intestinal *Acinetobacter* may increase energy harvest from food and promote silkworm growth [64]. Mishira et al. reported that *Acinetobacter* sp. strain BMW17 is a promising candidate for stimulating phytoremediation and degrading cellulosic waste [62]. Briones-Roblero et al. found that *Acinetobacter lwoffii* has lipolytic and esterase activity [65].

*Chryseobacterium* was higher in AD_I_ vs. MB_I_, which may be because AD has more crude protein than MB. *Chryseobacterium* strains are widely distributed in various natural environments such as water (including marine environments), soils, fish, and clinical specimens. It has strong keratinase or proteolytic activity and is used for feather degradation [5,66,67,68,69]. *Chryseobacterium*, as a plant growth-promoting rhizobacterial endophyte, was more abundant in intact roots than in rhizospheric or surrounding soil, due to its tendency to form biofilm [70]. Skowronek et al. found that *Chryseobacterium* had antagonistic activity against the pathogenic bacteria *Xenorhabdus* and *Photorhabdus* [71].

In addition, some bacteria, such as *Rhodococcus* and *Pandobacterium*, were positively correlated with cocoon quality, and although their abundance and importance based on random forests were lower than those mentioned above, they are also potentially important probiotic bacteria, and thus require further study to determine their functions. The bacteria that were positively correlated with cocoon quality or feed efficiency in AD_I_ vs. MB_I_ with relatively high abundance are shown in Figures S9 and S10, from which probiotic bacteria can be screened through further research.

There are no previous reports on the complexity of the interactions within silkworm intestinal microbial communities. Further research is needed to determine why the correlations in the network analysis were more complex for AD_I_ vs. MB_I,_ and why there were fewer intestinal microbiota OTUs on the 6th day even if the complexity of the interactions was higher on the 6th day. Dong et al. reported that the top KEGG level 1 functions of AD_I_ were Metabolism, Genetic Information Processing, and Environmental Information Processing [26]. This is consistent with our findings; i.e., these three functions were the top three in both AD_I_ and MB_I_. Further research is needed to determine why the cocoon quality was lower in AD_S_ vs. MB_S_, while the relative abundances of all six KEGG level 1 functions were significantly higher in AD_I_ vs. MB_I_. There are no previous reports on BugBase phenotype prediction analyses of silkworm intestinal microbiota, and further research is needed to determine why only Gram_Positive did not significantly differ between AD_I_ and MB_I_. Hormones play an important role in regulating insect growth and development [72,73], and microorganisms and hormones are also closely related to the host’s physiology and pathology [74]. Further research is also needed to determine why the cocoon quality was lower in AD_S_ vs. MB_S_, while the relative abundances of hormone-related functions were significantly higher in AD_I_ vs. MB_I_.

## 5. Conclusions

In this study, the differences in AD_I_ vs. MB_I_ (fifth instar) over time were analyzed. AD had a significant impact on the functions of the silkworm intestinal microbiota. The richness was significantly higher in AD_I_ vs. MB_I_. Proteobacteria was the most dominant bacterial phylum in MB, AD, and silkworm intestinal microbiota, regardless of sex, feed, and date. The most dominant bacterial genus in the silkworm intestinal microbiota differed by sex, feed, and date. Only a small proportion of intestinal bacteria are derived from the bacteria in feed. The correlations in the network analyses were more complex for ADI vs. MB_I_. Proteobacteria was positively correlated with cocoon shell rate and amounts of ingested and digested food. There were significant differences in all KEGG level 1 functions and all BugBase phenotypes in AD_I_ vs. MB_I_. The feed efficiency and cocoon quality of silkworms fed AD were affected by the diversity, community structure, and predicted functions of intestinal bacteria (Figure S11). This study provides data on AD_I_ and MB_I_ structure and changes over time, which may be used to develop probiotics and increase the conversion of raw materials (diet) into silk.

## Data Availability

All sequences have been deposited in the National Center for Biotechnology Information (NCBI) Sequence Read Archive database (accession no. PRJNA1128353).

## Supplementary Materials

The following supporting information can be downloaded at: https://www.mdpi.com/article/10.3390/insects15120970/s1, Table S1: PCR details; Table S2: Operational taxonomic unit richness and diversity indices of different samples; Table S3: Correlation network analysis of microbial communities; Table S4: Two way Correlation network analysis of microbial communities; Table S5: Functions of microbial communities on level 1; Table S6: The table of top relative abundance of genus among treatments; Figure S1: Silkworm and cocoon; Figure S2: Rarefaction curves depicting the number of OTUs with 97% similarity identified from different samples; Figure S3: Venn diagrams of the number of OTUs identified in different groups; (a) MB and AD. (b) MBI and ADI. (c) MB, MBI_f, and MBI_m. (d) AD, ADI_f, and ADI_m. (e) MB, MBI_1, MBI_4, and MBI_6. (f) AD, ADI_1, ADI_4, and ADI_6; Figure S4: Relative abundance of bacteria at the genus level in each sample. (a) MB and AD. (b) MBI and ADI. (c) MB, MBI_f, and MBI_m. (d) AD, ADI_f, and ADI_m. (e) MB, MBI_1, MBI_4, and MBI_6. (f) AD, ADI_1, ADI_4, and ADI_6; Figure S5: PCoA based on similarity of bacterial community composition among groups. (a) All groups. (b) MB and AD. (c) MB, MBI_f, and MBI_m. (d) AD, ADI_f, and ADI_m. (e) MB, MBI_1, MBI_4, and MBI_6. (f) AD, ADI_1, ADI_4, and ADI_6; Figure S6: Functions of microbial communities on level 1; Figure S7: Hormone-related functions of microbial communities on level 3; Figure S8: Main nutrient content; Figure S9: Bacteria that are positively correlated with cocoon quality or feed efficiency in ADI vs MBI; Figure S10: Bacteria that are negatively correlated with cocoon quality or feed efficiency in ADI vs MBI; Figure S11: Schematic model.
