# Supplementary material for: Dynamic Alterations of the Intestinal Microbiota of Fifth-Instar Silkworms (Bombyx mori) Fed an Artificial Diet or Mulberry Leaves
Source: Insects. 2024 Dec 5;15(12):970. doi: 10.3390/insects15120970 (PMC11677146; doi:10.3390/insects15120970)
Supplement: Supplementary file 1 [file insects-15-00970-s001.zip › insects-3328125-supplementary.pdf]

Supplementary Material

Table S1 PCR details

	experiment details
Main nutrient content	Available phosphorous (AP) was extracted from soil samples using 0.5 M $NaHCO_3$, and measured using a segmented continuous flow analyzer (Quaatro, Bran+Luebbe, Germany). 钙
PCR mixtures	The PCR mixtures contained 4 μL of 5x TransStartFastPfu buffer, 2 μL of 2.5 mM dNTPs, 0.8 μL of each primer (5 μM each), 0.4 μL of *TransStart*FastPfu DNA Polymerase, 10 ng template DNA, adding ddH_2O to a final volume of 20 μL. All reactions were performed in triplicate.
PCR cycling conditions	PCR cycling conditions included an initial denaturation at 95°C for 3 min, 27 cycles of denaturing at 95°C for 30 s, annealing at 55°C for 30 s, and extension at 72°C for 45 s, followed by a single extension at 72°C for 10 min and a continued hold at 4°C.
Splicing and quality control	Raw sequence reads were demultiplexed, quality-filtered by fastp version 0.20.0, and merged by FLASH version 1.2.7. Operational taxonomic units (OTUs), with a 97% similarity cut-off, were clustered using UPARSE v.7.1, and chimeric sequences were identified and removed. The taxonomy of each OTU representative sequence was analyzed by RDP Classifier v.2.2 against the 16S rRNA database using a confidence threshold of 0.7.

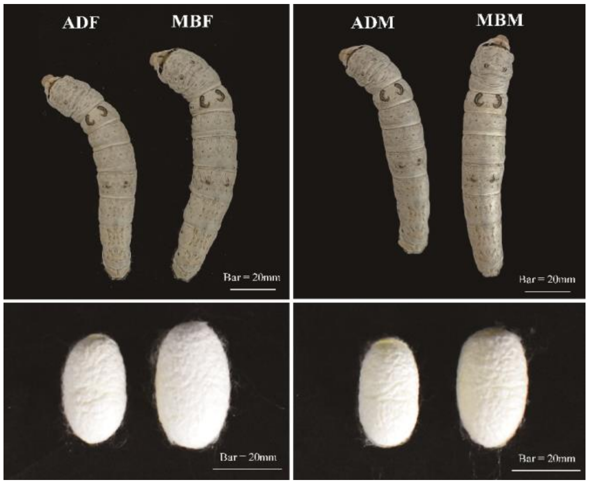

Figure S1 Silkworm and cocoon

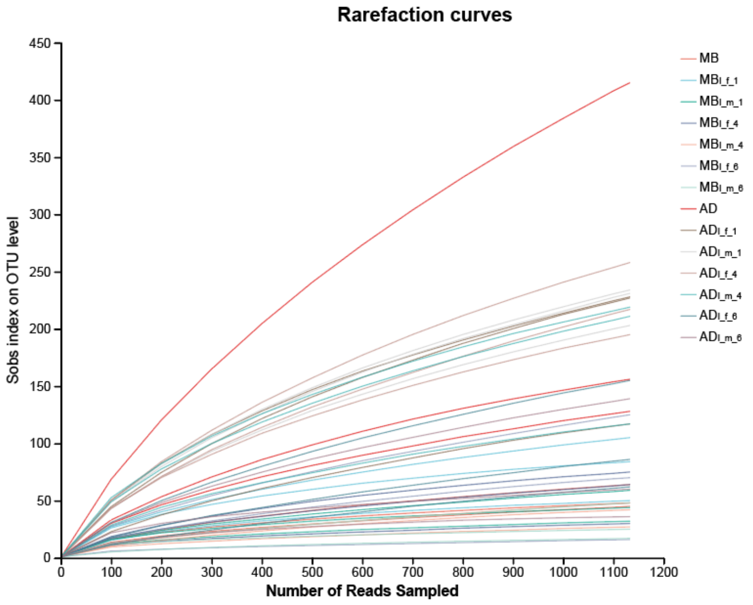

Figure S2 Rarefaction curves depicting the number of OTUs with 97% similarity identified from different samples

Table S2 Operational taxonomic unit richness and diversity indices of different samples

	sobs	shannon	simpson	ace	chao	coverage
AD	233a	3.83a	0.072e	603.3a	431.5ab	0.882e
$AD_{I\_f\_1}$	190.7ab	3.43ab	0.114de	432.4abc	310.4abc	0.913cde
$AD_{I\_m\_1}$	222.7a	3.93a	0.065e	514ab	367.3ab	0.9de
$AD_{I\_f\_4}$	223.3a	3.87a	0.07e	600.1a	450.1a	0.889de
$AD_{I\_m\_4}$	182.3ab	3.77a	0.069e	453.1abc	309.3abc	0.919bcde
$AD_{I\_f\_6}$	101bc	1.85cd	0.424abc	313.5abc	217.2bcd	0.946abcd
$AD_{I\_m\_6}$	78.3c	2.45bc	0.179de	188.5bc	124.9cd	0.969abc
MB	52.3c	1.93cd	0.291bcde	114.2c	80.8cd	0.979ab
$MB_{I\_f\_1}$	79.7c	2.46bc	0.208cde	152.8bc	119.5cd	0.97abc
$MB_{I\_m\_1}$	45c	1.65cd	0.408abc	115.1c	86.6cd	0.981a
$MB_{I\_f\_4}$	56.3c	1.81cd	0.328bcd	149.4bc	92.7cd	0.976ab
$MB_{I\_m\_4}$	39c	1.37cd	0.435ab	137.4c	80.6cd	0.981a
$MB_{I\_f\_6}$	70.3c	1.94cd	0.303bcd	231.4bc	140.8cd	0.964abc
$MB_{I\_m\_6}$	30c	1.12d	0.579a	183.2bc	63.5d	0.986a

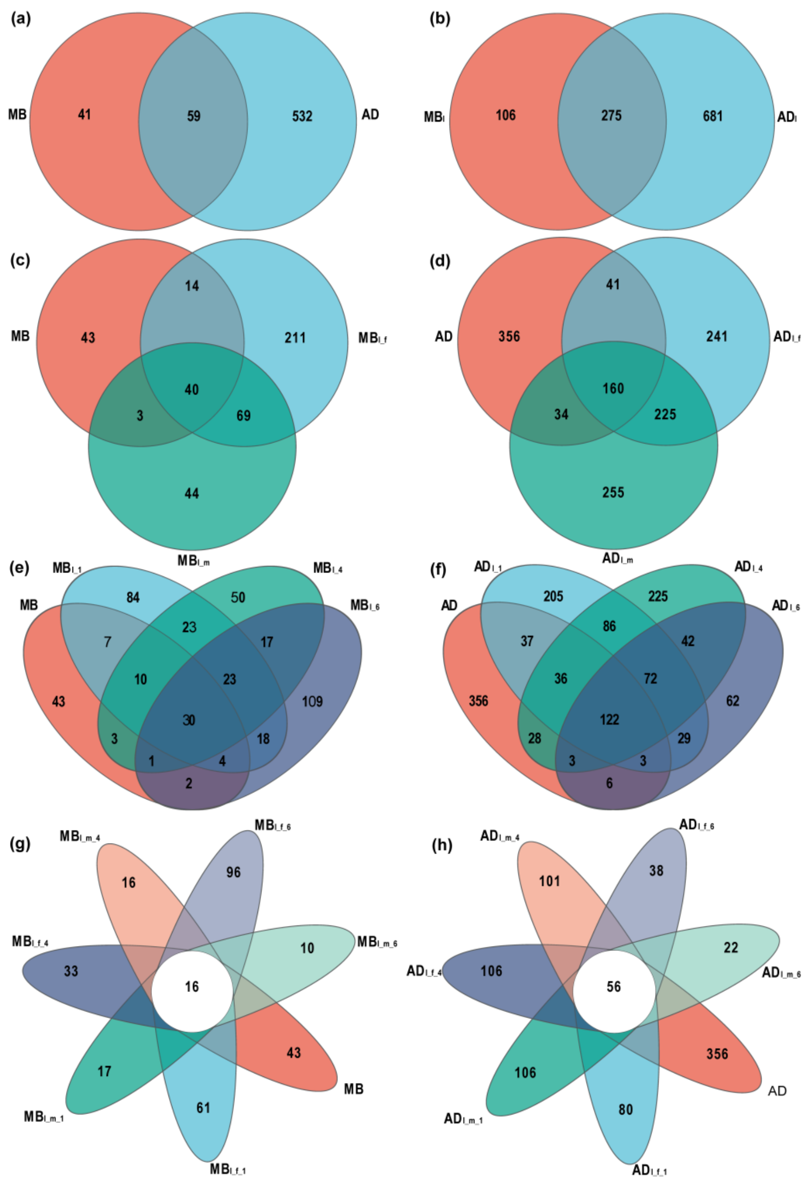

Figure S3. Venn diagrams of the number of OTUs identified in different groups

MB: mulberry leaves. AD: artificial diet. MB$_{I}$: intestinal microbiota of silkworms fed mulberry leaves. AD$_{I}$: intestinal microbiota of silkworms fed artificial diet. MB$_{I_f}$: intestinal microbiota of female silkworms fed mulberry leaves. MB$_{I_m}$: intestinal microbiota of male silkworms fed mulberry leaves. AD$_{I_f}$: intestinal microbiota of female silkworms fed artificial diet. AD$_{I_m}$: intestinal microbiota of male silkworms fed artificial diet. MB$_{I_1}$: intestinal microbiota of 1st-day silkworms of 5th instar fed mulberry leaves. MB$_{I_4}$: intestinal microbiota of 4th-day silkworms of 5th instar fed mulberry leaves. MB$_{I_6}$: intestinal microbiota of 6th-day silkworms of 5th instar fed mulberry leaves. AD$_{I_1}$: intestinal microbiota of 1st-day silkworms of 5th instar fed artificial diet. AD$_{I_4}$: intestinal microbiota of 4th-day silkworms of 5th instar fed artificial diet. AD$_{I_6}$: intestinal microbiota of 6th-day silkworms of 5th instar fed artificial diet. The following figures use the same abbreviations.

(a) MB and AD. (b) MB$_{I}$ and AD$_{I}$. (c) MB, MB$_{I_f}$, and MB$_{I_m}$. (d) AD, AD$_{I_f}$, and AD$_{I_m}$. (e) MB, MB$_{I_1}$, MB$_{I_4}$, and MB$_{I_6}$. (f) AD, AD$_{I_1}$, AD$_{I_4}$, and AD$_{I_6}$.

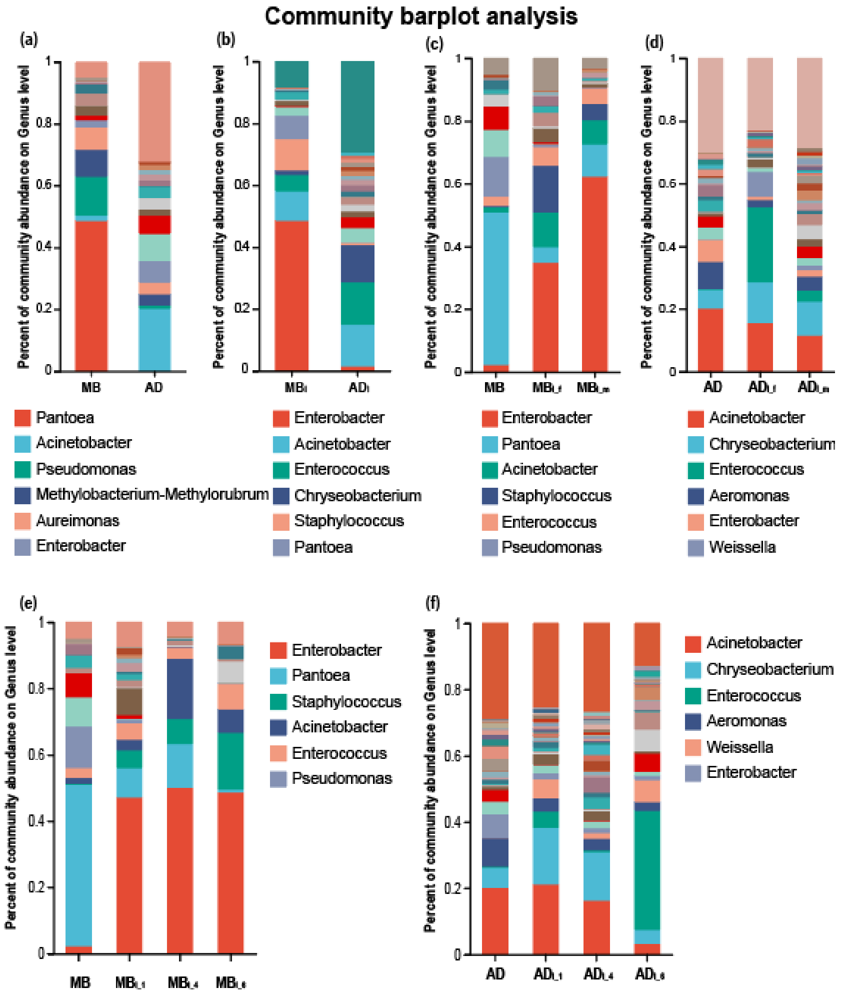

Figure S4 Relative abundance of bacteria at the genus level in each sample

(a) MB and AD. (b) MB_I and AD_I. (c) MB, MB_{I_f}, and MB_{I_m}. (d) AD, AD_{I_f}, and AD_{I_m}. (e) MB, MB_{I_1}, MB_{I_4}, and MB_{I_6}. (f) AD, AD_{I_1}, AD_{I_4}, and AD_{I_6}.

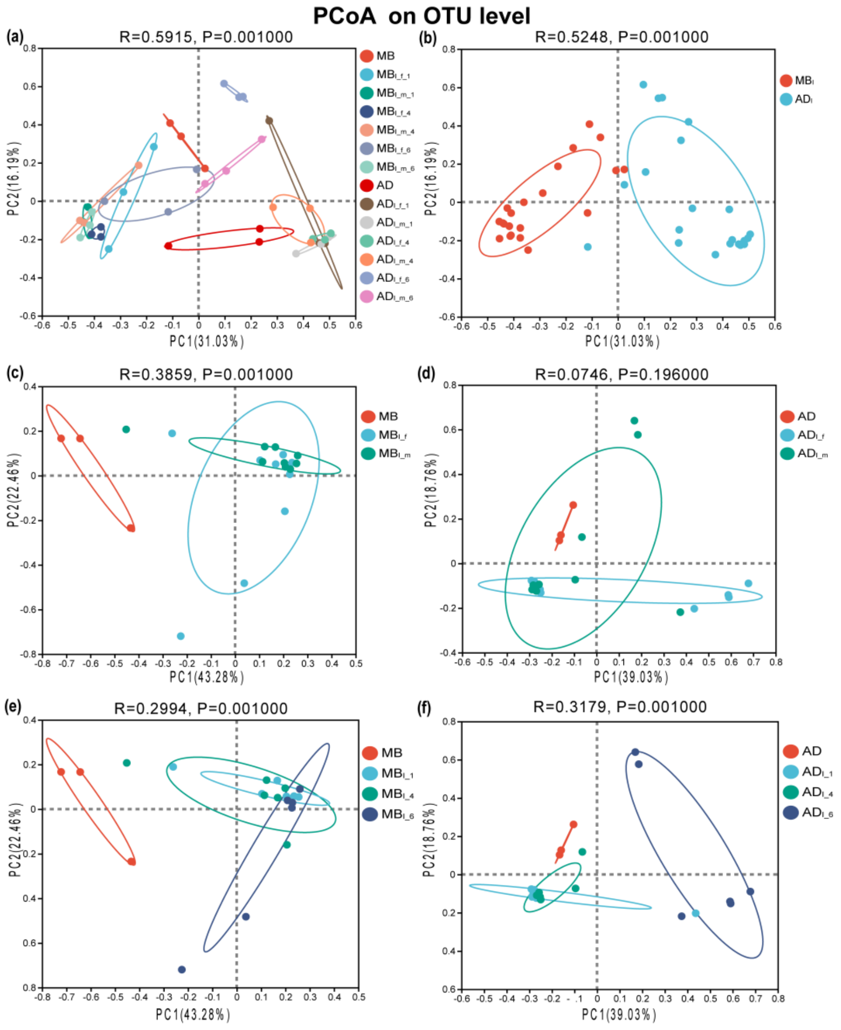

Figure S5. PCoA based on similarity of bacterial community composition among groups

(a) All groups. (b) MB and AD. (c) MB, MB_{I_f}, and MB_{I_m}. (d) AD, AD_{I_f}, and AD_{I_m}. (e) MB, MB_{I_1}, MB_{I_4}, and MB_{I_6}. (f) AD, AD_{I_1}, AD_{I_4}, and AD_{I_6}.

Table S3 Correlation network analysis of microbial communities

	Node numbers	Edge numbers	Node average degree	Positive edges	Negative edges
AD_{I_1}	25	37	2.96	31	6
AD_{I_4}	24	31	2.58	22	9
AD_{I_6}	22	81	7.36	46	35
AD_{I_f}	30	242	16.13	209	33
AD_{I_m}	28	68	4.86	57	11
AD_{I}	28	117	8.36	113	4
MB_{I_1}	27	45	3.33	38	7
MB_{I_4}	21	29	2.76	23	6
MB_{I_6}	24	50	4.17	49	1

MB_{I_f}	25	45	3.6	42	3
MB_{I_m}	21	47	4.48	40	7
MB_{I}	22	49	4.45	49	0

The correlation network indices were calculated based on the top 30 genera. The average number of connections per node in the network, that is, the node connectivity.

Table S4 Two way Correlation network analysis of microbial communities

Top 30	Node numbers	Edge numbers	Node average degree	Positive edges	Negative edges
AD_{I_1}	15	14	1.87	5	9
AD_{I_4}	25	29	2.32	23	6
AD_{I_6}	14	15	2.14	8	7
MB_{I_1}	22	24	2.18	12	12
MB_{I_4}	12	9	1.5	5	4
MB_{I_6}	7	4	1.14	4	0

The correlation network indices were calculated based on the top 30 genera. The average number of connections per node in the network, that is, the node connectivity.

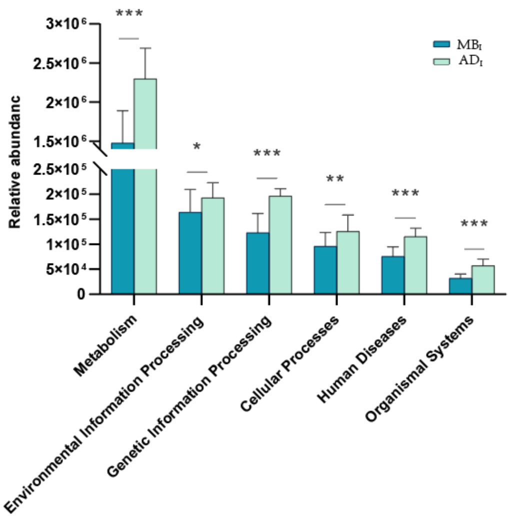

Figure S6 Functions of microbial communities on level 1

Table S5 Functions of microbial communities on level 1

	Metabolism	Environmental Information Processing	Genetic Information Processing	Cellular Processes	Human Diseases	Organismal Systems
$MB_{I\_f\_1}$	cd	abcd	bc	bc	bc	b
$MB_{I\_m\_1}$	d	cd	c	c	c	b
$MB_{I\_f\_4}$	d	d	c	c	bc	b
$MB_{I\_m\_4}$	bcd	ab	bc	ab	ab	b
$MB_{I\_f\_6}$	bcd	bcd	ab	c	bc	b
$MB_{I\_m\_6}$	d	cd	c	c	c	b

$AD_{I\_f\_1}$	abc	abcd	a	bc	a	a
$AD_{I\_m\_1}$	ab	abcd	a	ab	a	a
$AD_{I\_f\_4}$	abc	abcd	a	bc	a	a
$AD_{I\_m\_4}$	ab	abc	a	a	a	a
$AD_{I\_f\_6}$	abcd	ab	a	c	ab	b
$AD_{I\_m\_6}$	a	a	a	a	a	a

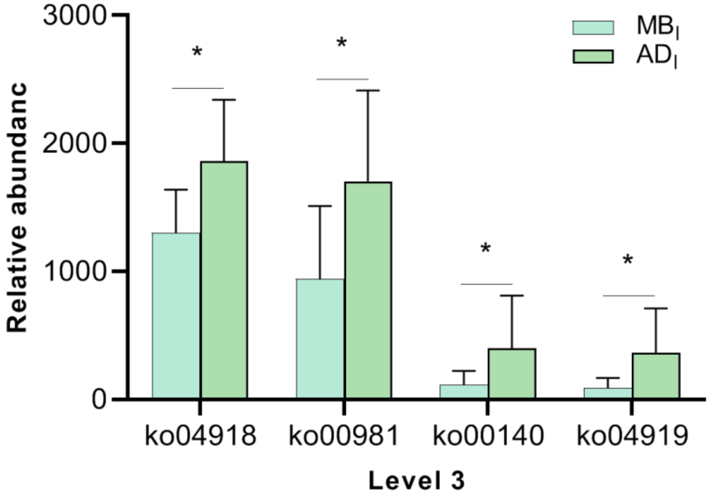

Figure S7 Hormone-related functions of microbial communities on level 3

Note: ko04918(Thyroid hormone synthesis), ko00981(Insect hormone biosynthesis), ko00140(Steroid hormone biosynthesis), ko04919(Thyroid hormone signaling pathway).

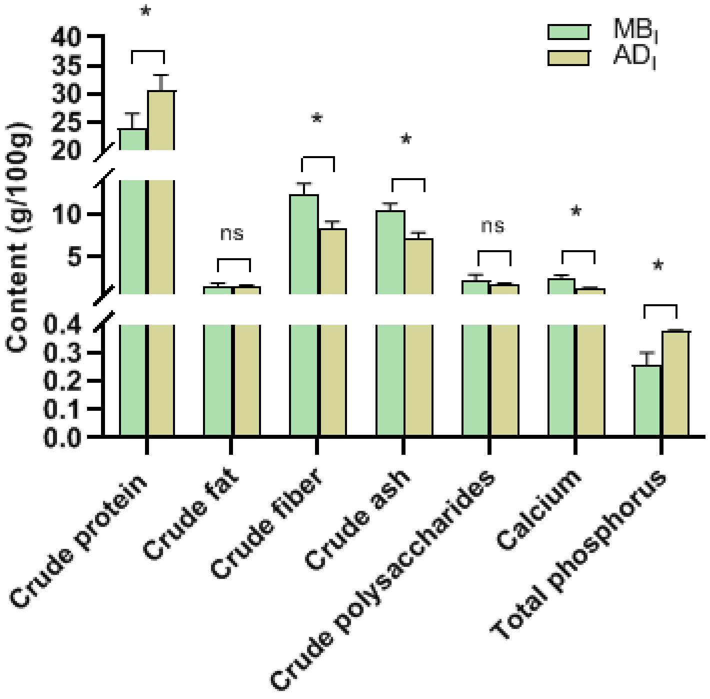

Figure S8 Main nutrient content

Table S6 The table of top relative abundance of genus among treatments

	Top 1		Top 2		Top 3	
	Genus	Relative abundance(%)	Genus	Relative abundance(%)	Genus	Relative abundance(%)
AD	Acinetobacter	20.12	Aeromonas	8.59	Enterobacter	7.12
AD $_{I\_f\_1}$	Acinetobacter	21.54	Chryseobacterium	16.24	Weissella	11.33
AD $_{I\_f\_4}$	Acinetobacter	20.45	Chryseobacterium	18.62	Haemophilus	6.35
AD $_{I\_f\_6}$	Enterococcus	62.05	Weissella	10.97	Acinetobacter	4.88
AD $_{I\_m\_1}$	Acinetobacter	20.83	Chryseobacterium	18.03	Aeromonas	6.06
AD $_{I\_m\_4}$	Acinetobacter	12.03	Chryseobacterium	10.65	Kerstersia	9.21
AD $_{I\_m\_6}$	Comamonas	13	Rhodococcus	10.91	Pandoraea	10.3
MB	Pantoea	48.66	Pseudomonas	12.59	Methylobacterium-Methylorubrum	8.77
MB $_{I\_f\_1}$	Enterobacter	31.69	Weissella	13.24	Pantoea	12.92
MB $_{I\_f\_4}$	Enterobacter	50.28	Acinetobacter	23.98	Staphylococcus	9.74
MB $_{I\_f\_6}$	Staphylococcus	30.71	Enterobacter	22.59	Glutamicibacter	11.92
MB $_{I\_m\_1}$	Enterobacter	62.58	Staphylococcus	6.83	Enterococcus	5.06
MB $_{I\_m\_4}$	Enterobacter	49.93	Pantoea	24.24	Acinetobacter	12.53
MB $_{I\_m\_6}$	Enterobacter	74.64	Acinetobacter	9.47	Enterococcus	6.21

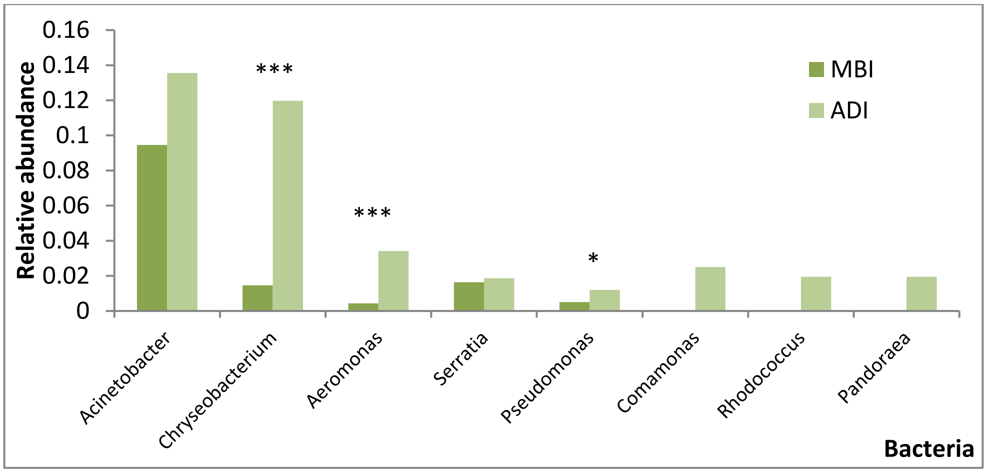

Figure S9 Bacteria that are positively correlated with cocoon quality or feed efficiency in AD_I vs MB_I

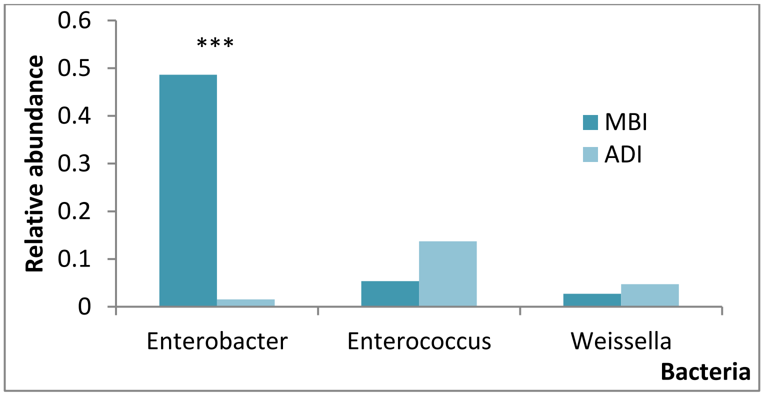

Figure S10 Bacteria that are negatively correlated with cocoon quality or feed efficiency in AD_I vs MB_I

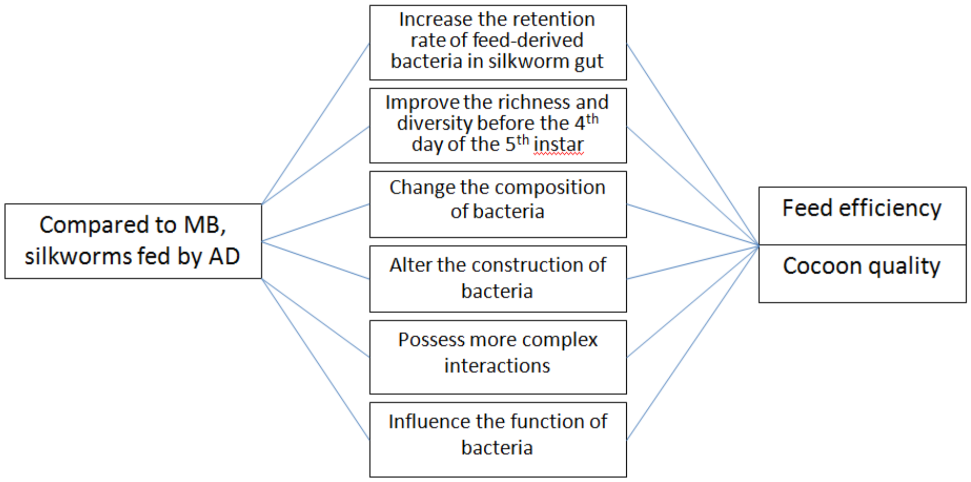

Figure S11 Schematic model
